# Dendritic Cell-Airway Epithelial Cell Cross-Talk Changes with Age and Contributes to Chronic Lung Inflammatory Diseases in the Elderly

**DOI:** 10.3390/ijms18061206

**Published:** 2017-06-06

**Authors:** Anshu Agrawal

**Affiliations:** Division of Basic and Clinical Immunology, Department of Medicine, University of California Irvine, Irvine, CA 92697, USA; aagrawal@uci.edu; Tel.: +1-949-824-7706

**Keywords:** dendritic cells, airway epithelial cells, age, inflammation, human, crosstalk

## Abstract

Age-associated dysregulated immune and inflammatory responses are one of the major factors responsible for the prevalence of chronic respiratory diseases in the older population. Pulmonary dendritic cells (DCs) are present below the airway epithelial cells (AECs) and are critical in initiating effective immune responses to harmful pathogens while maintaining tolerance against harmless antigens. The interaction between DCs and AECs plays a crucial role in lung immunity at homeostasis and during infections. The functions of both DCs and AECs are impacted with age. The present report reviews how the potential crosstalk between pulmonary DCs and AECs is dysregulated in the elderly impairing the capacity to maintain tolerance at the respiratory surfaces, which results in severe and chronic respiratory inflammatory diseases. We also discuss how such DC-AECs crosstalk will provide insight into the mechanisms underlying the increased susceptibility of the elderly to pulmonary inflammatory diseases.

## 1. Introduction

Chronic respiratory diseases are a major cause of health concern in the elderly [1,2,3,4]. In the US, alone, close to 24 million adults are estimated to have evidence of impaired lung function, indicative of chronic obstructive pulmonary disease (COPD), with most patients being 65 years or older [5]. An estimated 10 million others are diagnosed with chronic bronchitis, with the highest rate detected in people aged 65 years or older [6,7]. Furthermore, nearly 90% of the 4.7 million diagnosed with emphysema are 50 years or older [8]. The incidence of cancer and idiopathic pulmonary fibrosis is also significantly higher in older adults [3,9]. Respiratory infections in the aged population are also often severe and frequently require hospitalization [10]. The prevalence of chronic respiratory diseases is expected to double in the coming decades because of increased life expectancy in developed countries. Since the underlying pathological mechanisms of chronic respiratory diseases are not well understood, current therapies are neither specific nor effective. Airway immune cells are equipped to respond, and distinguish between, innocuous and pathogenic inhalants. One of the most important innate immune cells are dendritic cells (DCs), which act as initiators and regulators of the immune response [11]. DCs are present below the epithelial cell layer and survey the airway via extension of dendrites [12]. Emerging evidence indicates that airway DCs play a pivotal role in mediating airway tolerance against harmless antigens while mounting inflammatory responses against harmful pathogens [13,14,15]. This review focuses on the role of the interactions between DCs and airway epithelial cells (AECs) in chronic respiratory diseases of the elderly.

## 2. Age-Associated Deterioration and/or Dysregulation of Human Dendritic Cell Functions

Ageing has a significant impact on DC functions [16,17,18,19,20,21,22,23,24]. Several reports suggest that phagocytosis, migration, and cytokine secretion are impaired in DCs isolated from aged individuals (60–90 years of age) compared to DCs from young individuals (20–40 years of age) [25]. Moreover, we [17,26] and others [27] have shown that, even in the absence of infection, DCs from aged individuals exist in a semi-activated state that is characterized by an increase in the basal-level of Nuclear factor kappa B (NFκB) activation and pro-inflammatory cytokines production. We have also found that peripheral blood-derived DCs from aged individuals produce relatively higher basal levels of pro-inflammatory cytokines/chemokines Interleukin-6 (IL-6), C-X-C Motif Chemokine Ligand-8 (CXCL-8), CXCL-10, and Tumor necrosis factor-alpha (TNF-α), compared to young individuals [26]. In addition, DCs from aged individuals also produce high levels of metalloproteinases (known as ADAMs, or A Disintegrin And Metalloproteinase) as compared to DC from young individuals [26]. The ADAMs play an important role in tissue remodeling [28,29], via degradation of the structural proteins. This semi-activated state of aged DCs results in increased sensitivity to self-antigens, such as human DNA, and in the erosion of peripheral tolerance [17]. Furthermore, DCs from aged individuals, but not from young individuals, display an intrinsic defect in the production of interleukin-10 (IL-10), a potent anti-inflammatory cytokine that controls excessive production of pro-inflammatory mediators [20]. In addition, the secretion of protective cytokines in response to infections is impaired with aged DCs; DCs from aged individuals secrete reduced levels of Type-I-Interferons (IFNs) and Type III IFNs [18,19] in response to influenza and *Chlamydia pneumoniae* [30]. Other studies have also reported reduced secretion of type I IFNs by plasmacytoid dendritic cells (pDCs) and myeloid dendritic cells (mDCs) in response to viral infections [24,31,32,33]. While the role of type-I IFNs in viral protection is well established, emerging evidence indicates that type-III IFNs may play a more significant role in controlling the infections of the respiratory tract [34]. Respiratory viruses, such as influenza and respiratory syncytial virus, were reported to be more pathogenic and replicated to higher titers in the lungs of mice lacking receptors for both type-I and type-III IFNs compared to mice deficient in only type-I IFN receptor [35]. This is because receptors for type-I IFN, Interferon alpha receptor 1 (IFNAR1), and Interferon alpha receptor 2 (IFNAR2) are expressed on almost all tissues of the body while Interferon lambda receptor 1IFNLR1, one of the chains of the receptor for type-III IFNs, is expressed primarily on epithelial cells of the mucosa [34]. Impaired type-III IFN production against respiratory infections also enhances acute exacerbations, which are a major cause of morbidity and mortality in chronic respiratory diseases such as COPD [36]. The capacity of influenza-infected DCs from aged individuals to prime CD4+and CD8+ effector T cells is also significantly reduced, as compared to influenza-infected DCs from young individuals [18]. Deterioration and dysregulation of DCs’ function could contribute to age-associated elevated mucosal inflammation seen in the elderly. 

## 3. Age-Associated Changes in Airway Epithelial Cell (AEC) Functions

Ageing affects not only DC functions as outlined above, but also the functions of the AECs. One study reports that nasal epithelial tissue from aged subjects without respiratory disease displayed microtubular disarrangements and a significant reduction in ciliary beat frequency [37]. Ageing also decreased clearance of Teflon particles in small airways from older adults [38]. Furthermore, secretion of mucus by AECs is also affected with age. Mucus secretion, as measured by periodic acid-Schiff (PAS) staining and mRNA expression of mucin-5 subtype A and C (MUC-5AC), was found to be increased in older mice challenged intra-tracheally with ovalbumin [39]. A decrease in responses to oxidative stress was also observed in older AECs [40]. Age-associated changes in lung extracellular matrix components were also reported to affect the function of AECs. The expression of laminin α3, tissue factor, and N-cadherin was observed to be decreased in human bronchial epithelial cells incubated in old versus young lung matrix [41]. In a study of patients with chronic rhinosinusitis, age-associated reduced production of S100A8/9 proteins was observed in the elderly. S100A8/9 proteins or calprotectin are produced during infections and serve as chemoattractants for neutrophils and monocytes [42]. In summary, these studies suggest that AEC functions are significantly impacted with age and, thus, may play a major role in age-associated chronic respiratory diseases.

## 4. Immunological Cross-Talk between Airway Epithelial Cells (AECs) and Dendritic Cells (DCs) in Health and Disease

The epithelial cells lining the airways and nasal passages not only act as a barrier to prevent entry of the pathogens, but also play an active role in regulating immune responses. The close proximity of DCs to AECs results in continuous interaction and modulation of functions between the two cell types. For example, during viral infections DCs secrete pro-inflammatory cytokines, such as type I and type III interferons, which upregulate the expression of class I major histocompatibility complex (MHC) on AECs to enhance the antiviral responses [43]. The pro-inflammatory cytokines produced by DCs also act on tight junction proteins of the epithelial cell barrier to enhance the permeability and allow infiltration of immune cells to the site of infection [44]. As DCs influence epithelial cell functions, AECs can also affect the function of DCs [14,45]. AECs line the respiratory tract constituting the primary cellular barrier, expressing PRRs (pathogen recognition receptors) as well as receptors for allergens; this enables AECs to respond to antigens and allergens, thereby initiating the first step in the host-pathogen interaction [46]. 

Recent evidences suggest that local microenvironment defines the type of immune response elicited by the host [47]. Hammad et al. demonstrated that TLR4 signaling on AECs is responsible for the migration of mouse lung DCs to the mediastinal nodes in response to LPS inhalation [14]. In addition, viral infection of AECs also leads to the production of type I and III interferons, which activate DCs. Similarly activation of AECs by HDM (house dust mite) induces the release of various chemokines, such as C-C Motif Chemokine Ligand 2 (CCL2) and CCL20, which can attract immature DCs or monocytic precursors to the lung in mice [48,49]. DC-AEC interactions have also been demonstrated to play an important role in inducing Th2 responses against allergens [49]. Murine studies have indicated that activation of AECs by allergens induces the secretion of IL-25, IL-33, and thymic stromal lymphopoietin (TSLP). All of these act on DCs to enhance Th2 responses via OX40-OX40L interactions [49]. Recently, IL-25 has also been demonstrated to act on murine DCs to induce the secretion of CCL-17 which attracts Th9 cells during allergic inflammation [50]. However, in vitro human studies suggest that AECs may also prevent Th2 responses by enhancing the Th1 polarizing capacity of DCs since differentiation of monocytes in the presence of activated AECs leads to increased secretion of IL-12, IL-6, and TNF-α [51]. In addition to chemokines and cytokines produced by AECs, damage associated molecules or alarmins released during epithelial injury or death can also affect DC function. The concentration of alarmins, such as high mobility group box 1 protein (HM-GB1), Adenosine tri phosphate (ATP), and uric acid, are reported to be increased in the airways of asthmatic subjects. These molecules which are believed to be the products of dying epithelial cells can activate DCs to enhance inflammation [49]. 

In addition to affecting DC functions during infections, AECs also modulate DC function at homeostasis. The airways are constantly exposed to harmless, inhaled antigens and to prevent an immune response against them, the immune cells, such as DCs, have been reported to exist in a suppressed state, at least in mice [52]. AECs secrete retinoic acid (RA), Transforming growth factor-beta (TGF-β), and granulocyte macrophage colony stimulating factor (GM-CSF) in a steady state (in the absence of stimulation), rendering DCs and alveolar macrophages tolerogenic and prevent inflammation in the airways in murine studies [53,54,55]. Emerging evidence from human studies indicates airway monocytes have a more tolerant phenotype compared to blood monocytes, while lung CD1c+ DCs are more activated [56,57,58]. Baharom et al. [56] examined the phenotype of lung CD1c+ DCs in the airways and blood on samples from the airways via bronchial washing and broncho-alveolar lavage as well as mucosal tissue (endobronchial biopsies) from 20 healthy subjects with ages ranging from 18–40 years. They observed increased maturation of the CD1c+ DCs in the airway compared to blood and endobronchial biopsies. Another recent, comprehensive study by Granot et al. [58] has compared the myeloid DC subset distribution, maturation, and migration in mucosal tissues (lungs, intestines) and draining lymph nodes from 78 human organ donors with ages ranging from less than one year to 93 years. Their results suggest that highest numbers of CD1c+ DCs with migratory and mature phenotype are present in the lung draining lymph nodes. Furthermore, in another detailed study from 72 human lungs, pulmonary CD1c+ DCs were reported to be able to activate T cells in the mixed lymphocyte reaction [57]. In keeping with these observations, AECs have been shown to affect the immune surveillance function of DCs in a steady state. Using a 2D model system of monocyte-derived DCs and AECs, Rate et al. [59] observed an increased upregulation of several chemokine and TLR genes in DCs cultured with AECs at homeostasis. Our recent study also supports this data as we also observed upregulation of several pathogen recognition receptors (PRRs) and chemokine genes using a model of human myeloid DC (CD1c+ and CD141+) and AEC co-culture [60]. Furthermore, our study suggests that the CD1c+ subset of mDC was the major subset displaying the upregulation of PRR function while the CD141+ mDC subset was not affected. The above-mentioned studies with human lung [56,57,58] also did not report increased activation of the CD141+ mDC subset. Thus, it may be possible that there exists a division of labor between DC subsets with CD141+ mDC subset playing a role in tolerance and the CD1c+ mDC subset in immune surveillance. This suggests a scenario where AECs secrete factors to upregulate the immune surveillance function of CD1c+ DCs so that their response to infections is not compromised because of immunosuppressive environment of the airways. Collectively, these findings suggest that efficient interaction between the AECs and DCs contributes to respiratory health. Age-associated changes in the function of both AECs and DCs, thus, can prove detrimental to respiratory health.

## 5. Age-Associated Alterations in the Crosstalk between Airway Epithelial Cells and Dendritic Cells: Effect of DCs on AECs

**Figure 1:**
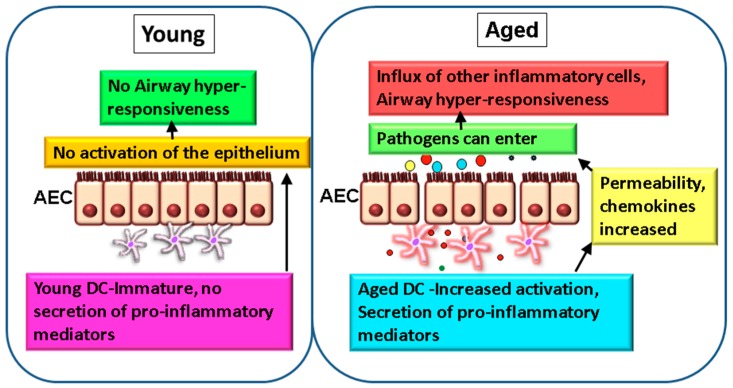
Altered DC-airway epithelial cell (AECs) crosstalk leads to impaired mucosal tolerance and inflammation in the elderly. In normal healthy lung from young subjects, dendritic cells (DCs) do not activate the epithelium at homeostasis and there is no inflammation and hyper-responsiveness (**left**). In contrast, in the aged lung, DCs secrete low levels of chronic inflammatory mediators, such as TNF-α and IL-6, which modulate the functions of AECs. The permeability, as well as secretion of chemokines (colored circles) by epithelium, are increased which allows infiltration of cells to the airways and leads to airway hyper-responsiveness. Permeability to infections is also increased (**right**).

While most studies have focused on murine models due the problems in obtaining lung tissue from humans, particularly from healthy aged subjects, few studies have examined the effect of age on mucosal DCs [61,62,63]. Biopsy of the lung is not a routine procedure and broncho-alveolar lavage (BAL) in the elderly is also not common. Furthermore, the numbers of DCs in the BAL are too few to perform mechanistic and functional studies. However, a very recent study by Granot et al. [58] has compared the DC populations in various organs including the lung, as a function of age. Though the authors did not observe a significant decline in DC numbers with age in all tissues, including the lungs, they did observe increased maturation of CD1c+ DCs in the mesenteric lymph nodes of older adults. Since ageing induces changes in the function of blood DCs, which also populate the lung, DC-AECs crosstalk may be affected. At homeostasis, virtually no information on the effect of DCs on AECs exists. To examine this and to simulate the airway mucosa, where DCs are in close contact with the epithelial cell barrier, we have developed a two-dimensional (2D) model where AECs are cultured on a transwell membrane insert and purified DCs from the blood are added to the bottom chamber. Such 2D culture systems are increasingly being used to replicate the lung environment and to study DC-epithelial crosstalk in humans [64,65]. As mentioned earlier, circulatory and monocyte-derived DCs from aged subjects displayed an enhanced basal state of activation [16,27]. The pro-inflammatory nature of DCs from aged subjects induced the activation of AECs in the absence of an acute insult (Figure 1) [26]. The barrier function of AECs was also compromised due to an increase in permeability. Furthermore, DCs from aged subjects induced significant upregulation of CXCL-10, CCL-20, and CCL-26 chemokines in AECs compared to DCs from young individuals. All three chemokines are involved in inflammation of the lung. CCL-20 (MIP3-β) is upregulated in the airways of patients with obstructive airway diseases [66], binding to CCR-6 expressed on epithelial cells and inducing the secretion of mucus. It also acts as a major chemoattractant for DCs [67]. CCL-26 (Eotaxin-3) attracts eosinophils, which are major players in lung inflammation [68]. CXCL-10 (IP-10) attracts T cells [69] and IP-10 levels are substantially increased in COPD patients [70,71]. The major factor responsible for the activation of AECs by DCs from aged individuals was the chronic, low-level TNF-α secretion. Altogether, this study suggests that aged DCs induce secretion of chemokines from AECs in the absence of any stimulation, attracting pro-inflammatory cells into airways of the elderly (Figure 1). Infiltration of immune cells in the airway will enhance inflammation. Furthermore, DCs from aged subjects also secrete increased levels of ADAMs, which are metalloproteases that can degrade the extracellular matrix components. In this context, ageing is associated with alterations to two extracellular matrix proteins: collagen and elastin [40,41]. 

## 6. Age-Associated Alterations in the Crosstalk between Airway Epithelial Cells and Dendritic Cells: Effect of AECs on DCs

**Figure 2:**
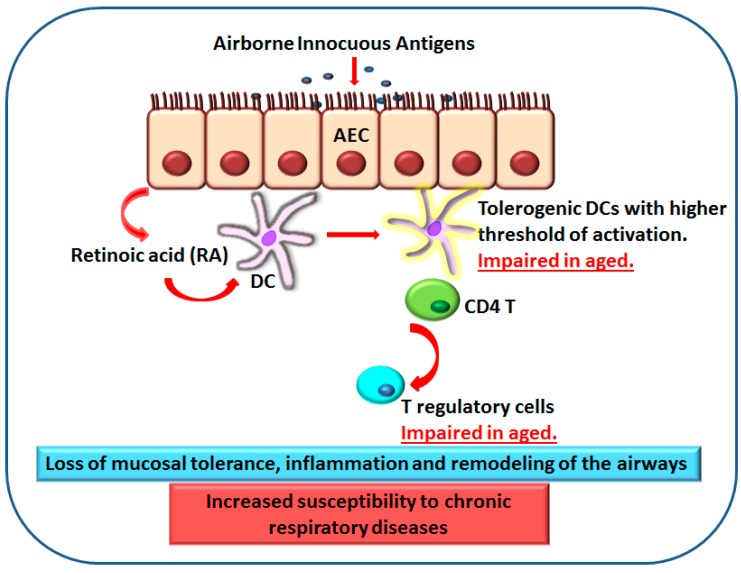
Impaired response of DCs from elderly to retinoic acid (RA) results in a loss of mucosal tolerance and inflammation. Retinoic acid produced by AECs (arrow 1, top) acts on DCs (arrow 2, middle) to induce T regulatory cells (arrow 3, bottom) and mucosal tolerance to prevent immune response to harmless inhaled antigens. DCs from aged subjects display reduced response to RA which leads to impaired induction of T regulatory cells and mucosal tolerance. This enhances inflammation and leads to airway hyper-responsiveness.

Airways are constantly exposed to harmless inhaled antigens. The DCs and macrophages in the airways exist in a state of immune suppression or tolerance to prevent immune response to the harmless antigens. Retinoic acid and other factors secreted by AECs play a crucial role in maintaining tolerance at these surfaces [72]. RA is a lipophilic molecule that controls the activity of a constellation of genes via binding to nuclear receptors [73,74,75]. Vitamin A is derived from the diet, and the liver constitutes a large reservoir of vitamin A in the form of retinal esters. Retinal esters are hydrolyzed to retinol and released into the blood [76]. Once retinol enters cells expressing appropriate enzymes, it is converted successively into retinal and RA [77]. The first step of the conversion is catalyzed by alcohol dehydrogenases and by microsomal retinol dehydrogenases (RDH) that are expressed by most cells, including DCs. The second step consists of the oxidation of retinal into RA and is catalyzed by three aldehyde dehydrogenases (ALDHs), known as retinaldehyde dehydrogenase 1 (RALDH1), RALDH2, and RALDH3, encoded by the *Aldh1a1*, *-2*, and *-3* genes, respectively [78]. RALDH expression is limited to certain cell types and, despite the widespread availability of retinol, only cells expressing one of the RALDHs can oxidize retinaldehyde to RA [73,74,79,80]. DCs, alveolar macrophages, and AECs at the mucosa are the three cell types that express RALDHs and can convert retinal to RA [45,80,81]. RA is essential for maintaining epithelial health and it also acts on DCs to render them tolerogenic and induce Foxp3^+^ regulatory T cells [73]. RA production and action by mucosa-associated DCs has been proposed to maintain the balance between effector T cells (Th1 and Th17 cells) and Foxp3+ regulatory T cells in the respiratory tract and to constitute a major mechanism underlying airway mucosal tolerance [81,82,83,84]. Ageing lung has been reported to display increased secretion of pro-inflammatory cytokines (IL-1β, IL-6, and TNF-α) at homeostasis suggesting a loss of mucosal tolerance [85]. Murine studies also indicate the presence of low grade chronic inflammation in the tracheas of older mice [86]. Insight into the possible mechanisms was obtained when the response of DCs from aged (>60 years) and young (20–40 years) subjects was examined for their response to RA. DCs from aged individuals displayed an impaired response to RA [87]. The induction of T regulatory cells was reduced by RA-treated DCs from aged subjects. The upregulation of two surface markers, CD141 and GARP (glycopherin associated regulatory protein) on DCs by RA was found to be essential for induction of T regulatory cells. The expression of both these surface receptors was reduced on the RA-treated DCs from aged individuals versus the young controls. Defective response to RA by aged DCs can, thus, enhance inflammation in the airways of the elderly (Figure 2). The status of production of RA by DCs and AECs during ageing has not been studied though age-associated decrease of vitamin A concentrations is well documented. Decreases in retinoid metabolism and signaling are also a characteristic of age-related disorders [88]. Thus, it is possible that reduced production, as well as decreased response of DCs to RA, impairs tolerance at the airways to enhance inflammation. In light of recent human studies mentioned earlier [56,57,58], it will be more interesting to determine the effect and production of RA on different mDC subsets especially since CD141+ mDCs are considered the human equivalent of CD103+ murine DCs, which is the subset most responsive to RA [53,89,90]. In addition to DCs, the response of alveolar macrophages to RA should also be examined as a murine study suggests that alveolar macrophages are the primary cells responding to RA and inducing regulatory T cells [81]. Furthermore, as mentioned, AECs also enhance the immune-surveillance function of DCs [59]; since DCs functions are compromised in the elderly, impaired surveillance may be the reason behind the age-associated increased susceptibility to respiratory infections. In addition, it will be important to determine the age-associated alterations in the status of IL-25, IL-33, and TSLP secretion by AECs as these may also play a significant role in COPD and asthma in the older adults. Virtually no information exists about these mediators in the elderly. 

## 7. Microbial Metabolites and DC Function in the Airways

In addition to AECs and DCs, the microbiome of the oral/airway can also affect DC and AEC function. Microbes secrete a number of metabolites that can act on cells to modulate their functions. For example, butyric acid produced by microbiota has also been demonstrated to play a major role in controlling inflammation in the gut [91]. In this respect, we have recently demonstrated that different microbes present in oral microbiome produce unique metabolites, which have a differential effect on the DC activation and T cell priming [92]. For instance, the *Pseudomonas* spp. secretes putrescine and glucose, which activate an inflammatory response in DCs, while *Streptococci* secrete 2,3-butanediol, which does not activate DCs. Interestingly, as people age, microbial communities in the oropharynx and anterior nares become more analogous, suggesting that ageing disrupts the ability to maintain distinct microbial communities at different sites [93]. Changes in the microbiome can affect the concentration of secreted metabolites which, in turn, can modulate the functions of immune cells to enhance inflammation. Alternatively, DCs from aged subjects may also display increased inflammatory responses to the microbial metabolites. Further studies are necessary to confirm these unanswered, interesting questions. 

## 8. Concluding Remarks

It is clear that DC-AEC interactions change with age at homeostasis. These changes can enhance airway inflammation by affecting the permeability and activation of the epithelial barrier. Furthermore, DCs from aged subjects also display a defect in responding to signals from epithelial cells, particularly RA which is essential for induction and maintenance of mucosal tolerance. This can further increase inflammation and remodeling in the airways. The aged airways may, therefore, be more permissible to cellular infiltrations as well as infections. Defects in DC-AEC crosstalk may, therefore, contribute to the age-associated increase in chronic respiratory diseases in the elderly.
